# NACK and INTEGRATOR act coordinately to activate Notch-mediated transcription in tumorigenesis

**DOI:** 10.1186/s12964-021-00776-1

**Published:** 2021-09-22

**Authors:** Elena Shersher, Mohini Lahiry, Annamil Alvarez-Trotta, Giulia Diluvio, David J. Robbins, Ramin Shiekhattar, Anthony J. Capobianco

**Affiliations:** 1 Molecular Oncology Program, Division of Surgical Oncology, Dewitt Daughtry Family Department of Surgery, University of Miami, 1600 NW 10th Ave, Miami, FL 33136 USA; 2 Sylvester Comprehensive Cancer Center, Miller School of Medicine, University of Miami, Miami, FL 33136 USA; 3 Cancer Epigenetics Program, Sylvester Comprehensive Cancer Center, Miller School of Medicine, University of Miami, Miami, FL 33136 USA; 4 Division of Surgical Oncology, Dewitt Daughtry Family Department of Surgery, Miller School of Medicine, University of Miami, Miami, FL 33136 USA; 5 Department of Human Genetics, Sylvester Comprehensive Cancer Center, Miller School of Medicine, University of Miami, Miami, FL 33136 USA

**Keywords:** Notch signaling, NACK, Integrator, Esophageal adenocarcinoma, Tumorigenesis, Transcriptional activation, SGK223

## Abstract

**Background:**

Notch signaling drives many aspects of neoplastic phenotype. Here, we report that the Integrator complex (INT) is a new component of the Notch transcriptional supercomplex. Together with Notch Activation Complex Kinase (NACK), INT activates Notch1 target genes by driving RNA polymerase II (RNAPII)-dependent transcription, leading to tumorigenesis.

**Methods:**

Size exclusion chromatography and CBF-1/RBPJ/Suppressor of Hairless/Lag-1 (CSL)-DNA affinity fast protein liquid chromatography (FPLC) was used to purify Notch/CSL-dependent complexes for liquid chromatography-tandem mass spectrometry (LC-MS/MS) analysis. Chromatin immunoprecipitation (ChIP) and quantitative polymerase chain reaction (qPCR) were performed to investigate transcriptional regulation of Notch target genes. Transfection of Notch Ternary Complex components into HEK293T cells was used as a recapitulation assay to study Notch-mediated transcriptional mechanisms. Gene knockdown was achieved via RNA interference and the effects of protein depletion on esophageal adenocarcinoma (EAC) proliferation were determined via a colony formation assay and murine xenografts. Western blotting was used to examine expression of INT subunits in EAC cells and evaluate apoptotic proteins upon INT subunit 11 knockdown (INTS11 KD). Gene KD effects were further explored via flow cytometry.

**Results:**

We identified the INT complex as part of the Notch transcriptional supercomplex. INT, together with NACK, activates Notch-mediated transcription. While NACK is required for the recruitment of RNAPII to a Notch-dependent promoter, the INT complex is essential for RNAPII phosphorylated at serine 5 (RNAPII-S5P), leading to transcriptional activation. Furthermore, INT subunits are overexpressed in EAC cells and INTS11 KD results in G2/M cell cycle arrest, apoptosis, and cell growth arrest in EAC.

**Conclusions:**

This study identifies the INT complex as a novel co-factor in Notch-mediated transcription that together with NACK activates Notch target genes and leads to cancer cell proliferation.

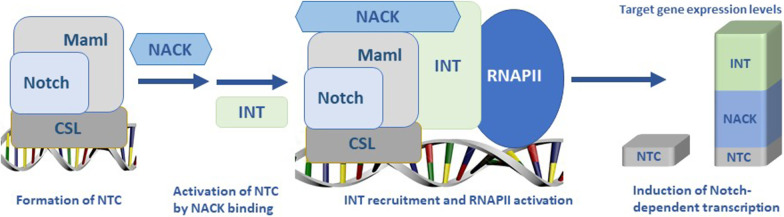

**Video abstract**

**Supplementary Information:**

The online version contains supplementary material available at 10.1186/s12964-021-00776-1.

## Background

Transcriptional regulation is an important and complex cellular process involving an integration of multiple signals via coordinated action of transcriptional co-factors. Many diseases including cancer are driven by abnormal cell signaling and deregulation of transcription. It is generally accepted that Notch functions as a transcriptional activator, and that aberrantly activated Notch signaling drives many aspects of the neoplastic phenotype [1–3]. However, the mechanism of how Notch regulates transcription remains unclear. Previously, we reported that NACK is a critical component in Notch1-driven transcription [4]. Notch-mediated transcription occurs when the Notch Intracellular Domain binds to the DNA-binding protein CSL and co-activator protein Mastermind-like (Maml) forming the Notch Ternary Complex (NTC) [5, 6]. Maml1 is then acetylated by p300 on residues Lys^188^ and Lys^189^ that leads to the recruitment of NACK to the NTC [7]. NACK is an atypical kinase and likely serves as a scaffold for the formation of a higher-order transcriptional complex and is required for the recruitment of RNAPII to target promoters [7–9]. These events in turn lead to the activation of a Notch transcriptional cascade that drives tumorigenesis.

Herein, we report that INT is a new component of the Notch transcriptional supercomplex that we define as the NTC bound to other transcriptional co-factors. INT is a multi-subunit complex consisting of at least 15 subunits with diverse biological functions such as production and processing of small nuclear RNA, biogenesis of enhancer RNAs, nucleic acid metabolism, development, and differentiation. A more recently discovered function of the INT complex is transcriptional regulation of protein-coding genes via interactions with RNAPII, although the specific nature of these interactions remains unclear. One of the major subunits of the INT complex is subunit 11 (INTS11) that has a metallo-β-lactamase domain and possesses endonucleolytic activity. INTS11 is part of the core cleavage module consisting of INTS4/INTS9/INTS11 and is required for efficient RNA processing. Furthermore, INTS11 has been shown to act as either a transcriptional repressor or activator depending on the gene class. Other INT subunits such as INTS13 and INTS8 have been implicated in regulation of enhancers and transcriptional elongation, respectively [10–15]. Structural modularity of the INT complex adds to its functional diversity and complicates the understanding of the INT role in biomolecular processes. Thus, a detailed knowledge of the transcriptional mechanisms mediated by INT is still lacking. In this study, we report that NACK and the INT complex act coordinately to initiate Notch1-mediated transcription by driving RNAPII recruitment and activation, respectively. Furthermore, inactivation of either NACK or INTS11 results in the abrogation of Notch-dependent transcription in cancer cells leading to cell growth arrest, and INTS11 knockdown results in G2/M arrest and apoptosis.

## Methods

### Cell culture and general reagents

Human T-cell lymphoma cell lines 4084 and 6780 were established in our laboratory and cultured as described previously [16]. Human EAC cell lines OE33 and OE19 were obtained from the European Collection of Cell Culture. Human EAC cell line SKGT2, immortalized esophageal epithelial cell line Het1A, and HEK293T cells were purchased from the American Type Culture Collection (ATCC). Human EAC cell line FLO1 was a generous gift from another lab [17]. Human EAC42 cell line was isolated from human esophageal mucosa obtained from tumor-adjacent tissue from a patient undergoing surgery at Miller School of Medicine, University of Miami. All EAC cell lines were maintained in RPMI 1640 supplemented with 10% fetal bovine serum, 100 units/mL penicillin, 100 µg/mL streptomycin, and 2 mM glutamine (complete RPMI). HEK293T cells were cultured in Dulbecco's Modified Eagle Medium (DMEM) supplemented with 10% fetal bovine serum, 100 units/mL penicillin, 100 µg/mL streptomycin, and 2 mM glutamine. Het1A cells were cultured as per ATCC instructions. OE33 spheres for animal experiments were cultured in DMEM/F12 medium supplemented with 1 µg/mL epidermal growth factor (Sigma, E5036), 1 µg/mL fibroblast growth factor (Sigma, F0291), and B27 supplement (Invitrogen, 17504-044). The number of the passages of the cell lines used in this study did not exceed 25. All cell lines were maintained at 37 °C in 5% CO_2_ and tested for mycoplasma contamination. The antibodies were purchased from Cell Signaling Technology (CST), Abcam, Thermo Fisher Scientific, Bethyl Laboratories, or Santa Cruz Biotechnology. All other chemicals were purchased from VWR unless stated otherwise.

### Purification and mass spectrometry analysis of Notch complexes

Notch1/CSL-dependent complexes were purified from nuclear extracts prepared from a Notch-driven T-cell lymphoma 4084 as described previously [16, 18]. Briefly, nuclear lysates were fractionated by size exclusion chromatography and a fraction with the higher-molecular-weight Notch-containing complexes was purified by CSL-DNA affinity FPLC. The CSL-DNA column contained oligonucleotides derived from high-affinity Notch transcriptional complex binding sites on the promoter of *HES1*, which is a canonical Notch target gene. The Notch complex was eluted in a stepwise manner with increasing salt concentration. The analysis of the eluates by western blot (WB) was performed to identify a fraction containing the NTC components Notch1, Maml1, and CSL. This fraction was analyzed by LC-MS/MS. A Myc-driven T-cell lymphoma cell line 6780 served as a negative control since there is no Notch1 or Maml1 expression in 6780 cells.

### ChIP-qPCR assay

The ChIP assay was performed as described previously [4]. Briefly, cells were crosslinked with 1% formaldehyde and chromatin fragments of approximately 200–800 bp were prepared by sonication. Chromatin samples were immunoprecipitated with the appropriate antibodies. DNA was purified using the PCR purification kit (Qiagen) and target sequences were amplified by qPCR. The following primer sequences were used for *HES1* and *HES5* promoters:

- *HES1* promoter: 5′-CGTGTCTCCTCCTCCCATT-3′ and 5′-GGGGGATTCCGCTGTTAT-3′.
- *HES5* promoter: 5′-GGGAAAAGGCAGCATATTGA-3′ and 5′-CACGCTAAATTGCCTGTGAA-3′.

For *HES4* and *CCND1* promoters, we used SimpleChIP promoter primers #7273 and #12531 from CST. Antibodies used in this study can be found in Additional file 1: Table S1.

### Cell transfections

The specific Silencer Select small interfering RNAs (siRNAs) targeting human INTS11 and a corresponding negative control siRNA were purchased from Thermo Fisher Scientific (Assays s29893, s29894, s29895, AM4635). The specific siRNA targeting human NACK and a corresponding negative control siRNA were purchased from Santa Cruz Biotechnology (sc-77433, sc-37007). A LipoJet In Vitro Transfection Kit was purchased from SignaGen Laboratories. The day before transfection, cells were seeded in cell culture plates containing complete RPMI medium and incubated for 24 h. The following day, transfection was performed using siRNAs and the LipoJet In Vitro Transfection Kit according to the manufacturer's instructions. The final concentrations of INTS11 and NACK siRNAs were 60 nM and 100 nM, respectively. The cells were collected either 72 h or 96 h after transfection.

pcDNA plasmids encoding human Notch1 (0.1 µg), Maml1 (0.01 µg), or NACK (0.01–0.1 µg) were used to transfect HEK293T cells. Transfection was performed as described for EAC cells with the following modifications. Complete DMEM medium was used for HEK293T cells and cell were collected 48 h after transfection.

### Viral infections

pLKO plasmids containing short hairpin RNA (shRNA) against INTS11 or green fluorescent protein (GFP) (as a control) was a kind gift from Dr. Shiekhattar’s lab. Lentivirus was packaged using psPAX2 packaging vector and pMD2.G envelope plasmid in HEK293T cells. HEK293T transfection was performed as described above. Virus was collected 48 h after transfection. For OE33 cells infection, virus-containing medium containing 4 µg/mL polybrene (Sigma, TR-1003-G) was added overnight. Infected cells were selected with either 2.0 µg/mL puromycin (Thermo Fisher Scientific, AAJ672368EQ) (shGFP cells) or 400 µg/mL geneticin (Thermo Fisher Scientific, 10131035) (shINTS11 cells). To address clonal variation, the knockdown of INTS11 was determined in various clones by RT-qPCR and is shown in Additional file 2: Fig. S1. shINTS11 OE33 clones 1 and 2 were chosen for our experiments.

### RT-qPCR analysis

Total mRNA was isolated using the RNeasy Plus Mini Kit (Qiagen) and cDNA was synthesized using the High-Capacity cDNA Reverse Transcription Kit (Applied Biosystems) following the manufacturer's directions. Reverse transcriptase quantitative polymerase chain reaction (RT-qPCR) analysis was performed using the TaqMan™ Universal Master Mix II (no UNG) and TaqMan™ Gene Expression Assays according to manufacturer’s instructions (Applied Biosystems). Gene expression was normalized to hypoxanthine phosphoribosyltransferase 1 (HPRT1) gene. TaqMan™ Gene Expression Assays used in the study are as follows: INTS11 (Hs00259909m1), CCND1 (Hs05042293s1), NOTCH3 (Hs01128537m1), NACK (Hs00410725m1), HES1 (Hs01118948g1), TBP (Hs00427620m1), ACTB (Hs00357333g1), HPRT1 (Hs02800695m1).

### Western blotting

Cells were lysed using radioimmunoprecipitation assay (RIPA) buffer. Cell lysates were resolved by sodium dodecyl sulphate–polyacrylamide gel electrophoresis and transferred onto nitrocellulose membranes using the Trans-Blot Turbo Transfer System (Bio-rad) following manufacturer’s instructions. Membranes were blocked in 5% milk and incubated with appropriate antibodies followed by incubation with an anti-mouse or anti-rabbit secondary antibody conjugated with horseradish peroxidase. The list of antibodies can be found in Additional file 1: Table S1. For detection, enhanced chemiluminescence reaction (Amersham Biosciences) was performed according to the manufacturer’s specifications.

### Colony formation assay

OE19, FLO1, and Het1A cells were transfected with siRNA as described above and cultured for 6–8 days. OE33 cells containing shRNA were cultured at low density under 1 µg/mL doxycycline treatment for 10 days. Colonies were fixed with methanol and stained with 0.01% crystal violet.

### Flow cytometry

OE19 cells were transfected with control siRNA and siRNA against INTS11 or Notch1 as described above for 72 and 96 h. To evaluate cell viability and apoptosis, cells were prepared using an Alexa Fluor® 488 Annexin V/Dead Cell Apoptosis Kit (Thermo Fisher Scientific) following manufacturer’s instructions. To evaluate a cell cycle, cells were prepared using a Propidium Iodide Flow Cytometry Kit (Abcam, ab139418). Then cells were passed through a cell strainer (Corning) and analyzed using BD LSRII flow cytometer.

### Animal experiments

Immunocompromised nude female mice with body weight ranging from 19 to 24 g were purchased from Charles Rivers Laboratories. Animals were maintained in a pathogen-free environment on a 12-h light/12-h dark cycle with food and water supplied ad libitum throughout the experimental period. To obtain a statistical significance of *p* < 0.05 and 85% probability (The Laboratory Animal Services Centre Sample Size Calculator was used [19]), mice were divided into 3 groups with 6 mice in each group. Groups 1 and 2 were inoculated with shINTS11 OE33 spheres (2 different clones) and group 3 was inoculated with shGFP (control) OE33 spheres. When tumors reached the size > 200 mm^3^, all mice were placed on a doxycycline diet (625 mg/kg) for 3 weeks. Tumor volume was measured by the formula: volume = (S × S × L)/2 [20].

### Statistical analysis

Statistical significance was determined by 2-tailed Student’s T test and data are presented as mean ± SD. *p* value < 0.05 was considered statistically significant.

## Results

### Integrator is part of the Notch transcriptional supercomplex

**Fig. 1:**
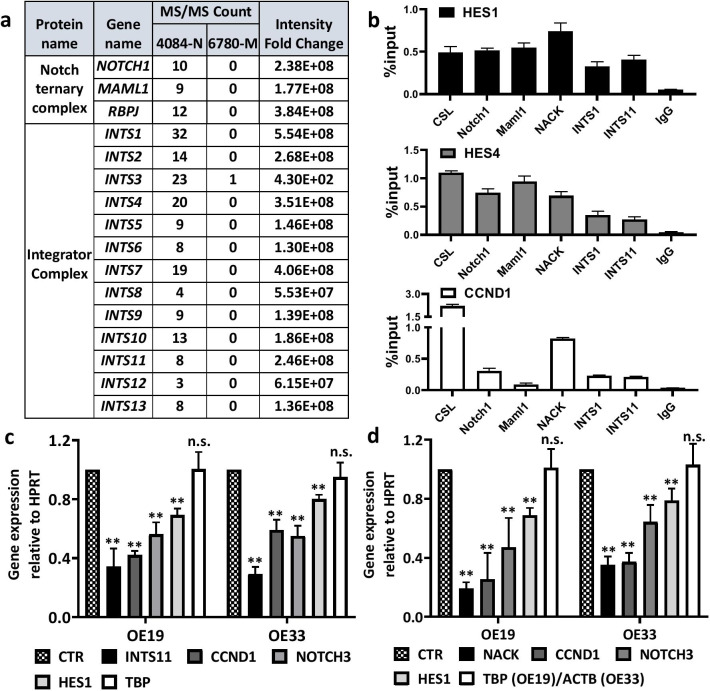
Integrator together with NACK activates Notch-mediated transcription. **a** MS/MS analysis shows that subunits of the Integrator complex co-purify with the Notch Ternary Complex in the Notch-driven T-cell lymphoma cell line 4084 by affinity chromatography. Notch-independent lymphoma 6780 was used as a negative control. **b** Representative ChIP on the *HES1, HES4,* and *CCND1* promoters in OE19 EAC. Bars represent standard deviation (N = 3). **c** Expression of Notch1 target genes in OE19 and OE33 upon INTS11 knockdown. Bars represent standard deviation (the mean from 3 biological replicates). **p < 0.01 versus CTR sample. **d** Expression of Notch1 target genes in OE19 and OE33 upon NACK knockdown. Bars represent standard deviation (the mean from 3 biological replicates). **p < 0.01 versus CTR sample

To investigate the mechanism of Notch transcriptional regulation, we sought to identify key downstream components that link the NTC to activation of transcription. Therefore, we purified Notch1/CSL-dependent complexes from nuclear extracts prepared from a Notch-driven T-cell lymphoma cell line (4084). Nuclear lysates were fractionated by size exclusion chromatography and the fraction with Notch1-containing complexes was purified by CSL-DNA affinity FPLC. An isogenic Myc-driven T-cell lymphoma cell line (6780) served as a negative control for purification since it lacks activated Notch and Maml. Eluate fractions containing Notch were subjected to LC-MS/MS to determine the identities of co-eluting proteins (Fig. 1a). This analysis revealed that we had co-purified 13 out of 15 known components of the Integrator complex with Notch, as they were not identified in the control cell line using the same purification scheme. These data indicate that INT is a component of the Notch transcriptional supercomplex and plays a role in Notch-mediated transcription.

In order to validate the results obtained from the purification and MS analysis in a system relevant to human cancer, we performed ChIP assays in Notch-dependent EAC cell lines OE19 and OE33. These cell lines were classified as Notch-dependent because they exhibit an inhibition of the cell growth as well as decreased viability and transcription of Notch target genes when treated with DAPT, which is a gamma-secretase inhibitor that inhibits Notch activity [21, 22]. Using Integrator subunits 1 and 11 (INTS1—the largest core subunit and INTS11—a catalytic subunit) as indicators of the INT complex in the ChIP analysis, we investigated the occupancy of INT on the *HES1, HES4*, and *CCND1* promoters (Fig. 1b and Additional file 3: Fig. S2). We observed both INTS1 and INTS11 ChIP signals on the promoters in both cell lines together with ChIP signals of CSL, Notch1, Maml1, and NACK. This result indicates that INT co-localizes with both the NTC and NACK on Notch target genes. Therefore, we validated the purification and MS analysis in the Notch-dependent tumor cells.

### Both INT and NACK are critical for transcriptional activation mediated by Notch

Previously, we have reported that the protein NACK plays an essential role in Notch-mediated transcriptional regulation. NACK is recruited to the NTC following the acetylation of Maml1 on two residues Lys^188^ and Lys^189^ by p300. Both p300 activity and wild-type Maml are required for the function of NACK [7]. Moreover, we have discovered that NACK is required for the recruitment of RNAPII to Notch-activated promoters. However, the mechanism of RNAPII recruitment by NACK is unclear. Since we observed INTS1 and INTS11 co-localization with NACK on the Notch target promoters, we explored the relationship between NACK and INT in Notch-mediated transcription. We depleted NACK or INTS11 in OE19 and OE33 EACs via RNA interference and analyzed transcription of several key Notch1 target genes (*CCND1*, *NOTCH3*, and *HES1*). The depletion of either INTS11 or NACK resulted in the attenuation of transcription of Notch target genes. When INTS11 was knocked down in both OE19 and OE33 cells via RNA interference, transcription of canonical Notch target genes was significantly reduced (Fig. 1c). Similarly, when NACK was knocked down by siRNA, we observed a reduction of transcription of a similar set of genes (Fig. 1d). These results indicate that NACK and INT are both critical for transcriptional activation mediated by Notch.

### NACK is required for Notch1, Integrator, and RNAPII on the Notch target promoter

**Fig. 2:**
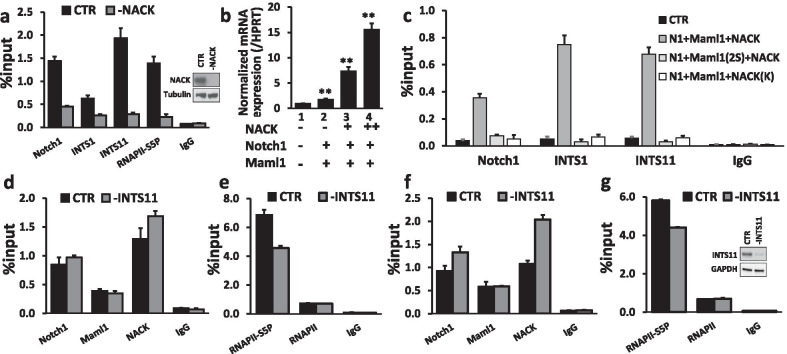
Integrator and NACK are required for the activated form of RNAPII phosphorylated at Ser5 (RNAPII-S5P) and other transcriptional co-factors on the Notch-dependent promoter. **a** Representative ChIP shows that the loss of NACK results in the decrease of Notch1, INTS1, INTS11, and RNAPII-S5P, required for transcriptional elongation, on the *HES1* promoter. Bars represent standard deviation (N = 3). WB shows depletion of NACK **b** In HEK293T cells, NACK activates *HES5* transcription in a NACK concentration-dependent manner. Bars represent standard deviation (N = 3). **p < 0.01 versus CTR sample. **c** Representative ChIP shows that in HEK293T cells expressing mutant Maml1 (Maml1(2S)) or mutant NACK (NACK(K)), NACK is unable to bind the NTC, thus *HES5* is not activated due to the absence of Notch1 and INT complex (INTS1, INTS11) on the promoter. Bars represent standard deviation (N = 3). **d** Representative ChIP shows that the knockdown of INTS11 does not affect the occupancy of Notch1, Maml1, and NACK on the *HES1* promoter. Bars represent standard deviation (N = 3). **e** Representative ChIP shows that the knockdown of INTS11 results in a decrease of RNAPII-S5P, but not unphosphorylated RNAPII, on the *HES1* promoter. Bars represent standard deviation (N = 3). **f** Representative ChIP shows that the knockdown of INTS11 does not affect the occupancy of Notch1, Maml1, and NACK on the *HES4* promoter. Bars represent standard deviation (N = 3). **g** Representative ChIP shows that the knockdown of INTS11 results in a decrease of RNAPII-S5P, but not unphosphorylated RNAPII, on the *HES4* promoter. Bars represent standard deviation (N = 3). WB shows depletion of INTS11

In order to investigate the relationship between NACK and other co-factors on a Notch-dependent promoter, we depleted NACK via RNA interference in OE33 and analyzed the occupancy of Notch1, the active form of RNAPII phosphorylated at Serine 5 (RNAPII-S5P), INTS1, and INTS11 on the *HES1* promoter via a ChIP assay. When NACK was depleted, we observed a profound decrease of RNAPII-S5P ChIP signal on the *HES1* promoter (Fig. 2a), in line with previously published data [7]. Similarly, the occupancy of Notch1, INTS1, and INTS11 on the *HES1* was also decreased when NACK was depleted, which indicates that NACK is important for the stabilization of the NTC and localization of INT to the promoter (Fig. 2a).

Since NACK is required for the presence of INTS11 and RNAPII-S5P on the promoter, we reasoned that an inability of NACK to bind the NTC should result in the absence of INTS1, INTS11, and RNAPII-S5P on the Notch target promoter, which should lead to the transcriptional block. To test this hypothesis, we assayed the reconstitution of the Notch transcriptional supercomplex in HEK293T cells by transfecting pcDNA3 vectors encoding various combinations of the NTC proteins. HEK293T cells express INTS1, INTS11, and CSL, but lack endogenous Notch, Maml, and NACK, thus allowing us to program the cells with various protein components of the NTC as wild-type (WT) or mutant versions and assess the activity. When cells were transfected with Notch1 and Maml1, there was a minimal induction of *HES5* transcription as compared to the cells transfected with an empty pcDNA vector (Fig. 2b, lanes 1 and 2). However, when NACK was added to the transfection, we observed a dramatic dose-dependent increase in *HES5* transcription (Fig. 2b, lanes 3 and 4). This result established the HEK293T recapitulation assay as a bone fide model for Notch-mediated transcription. We next sought to examine the effect on Notch-mediated transcription when we utilized previously described mutants of Maml1 and NACK that attenuate Notch-mediated transcription. Maml1(2S) harbors the K188R/K189R mutations that are no longer sites of acetylation. This mutant version results in the inability of p300 to acetylate Maml1 and thus prevents binding of NACK to the NTC [7]. The NACK(K) mutant possesses a K1002A mutation in a critical active site residue that renders NACK unable to bind the NTC [7]. To establish a baseline for the occupancy of Notch1 and INTS11 on the *HES5* promoter, HEK293T cells were transfected with Notch1, Maml1 and NACK, and the occupancy of Notch1, INTS1, and INTS11 was determined by ChIP analysis. The ChIP assay revealed that the Notch1, INTS1, and INTS11 occupancy on the *HES5* promoter substantially increased over the control condition (pcDNA3-transfected) (Fig. 2c). When WT Maml1 was substituted with the K188R/K189R (Maml1(2S)) mutant, there was a complete loss of Notch1, INTS1, and INTS11 binding. The same result was obtained when WT NACK was replaced with the K1002A mutant (NACK(K)). Both mutants prevent NACK from binding the NTC, which results in the absence of Notch1 and INT on the promoter. In turn, this leads to the loss of RNAPII-S5P on the *HES5* promoter as we have shown previously [7]. Together these data indicate that NACK is essential for the stabilization of the NTC, subsequent recruitment of INT and RNAPII to the Notch target promoter, and thus activation of transcription.

### Integrator is required for RNAPII-S5P, SPT5, and cyclin T1 on the Notch target promoter

Similarly, we examined the effect of INTS11 depletion on transcriptional co-factors on the *HES1* and *HES4* promoters in OE33. When INTS11 was depleted, there was no decrease in the occupancy of Notch1, Maml1, or NACK on both promoters indicating that INT is not required for the stabilization of the NTC or recruitment of NACK to the Notch-dependent promoters (Fig. 2d, f). In contrast, we observed a decreased occupancy of the active phosphorylated form of RNAPII (RNAPII-S5P), but not unmodified RNAPII, on both *HES1* and *HES4* promoters (Fig. 2e, g). This result indicates that INT is not required for the recruitment of RNAPII, but for its activation on the Notch target promoters. In addition, we evaluated the occupancy of SPT5 and Cyclin T1 (CCNT1) on the promoter of *HES1* when INTS11 was knocked down. These co-factors are known to interact with RNAPII and the INT complex and are involved in the initiation of transcription. CCNT1 is a subunit of the positive transcription elongation factor b (P-TEFb) needed for the elongation phase in transcription. SPT5 is part of DRB sensitivity-inducing factor (DSIF) that can act either as a negative or positive elongation factor depending on its phosphorylation status by P-TEFb [23–26]. With INTS11 depletion, we observed a decrease in the occupancy of both SPT5 and CCNT1 on the promoter indicating INT requirement for these co-factors (Additional file 4: Fig. S3).

### Integrator is over-expressed in Notch-dependent esophageal adenocarcinoma and it is required for EAC cell growth and tumorigenesis

**Fig. 3:**
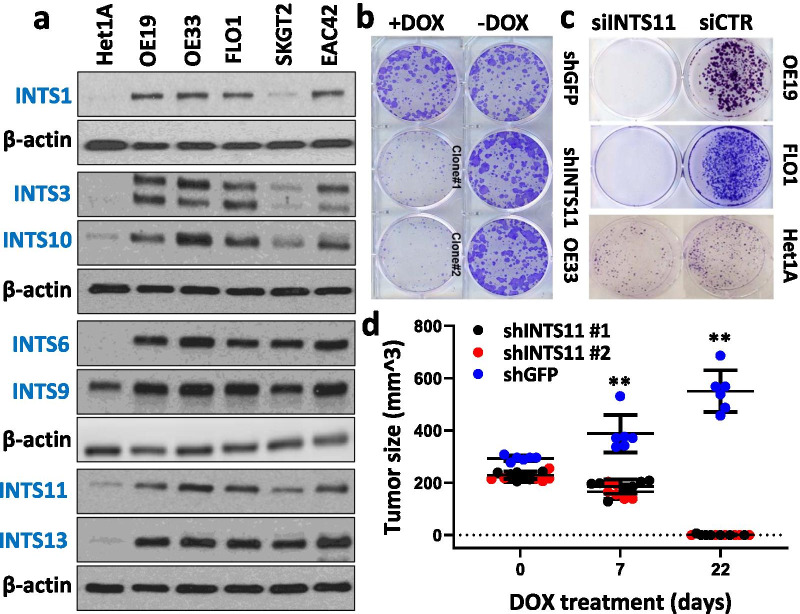
INT is required for Notch-dependent cell growth and tumorigenesis in EAC. **a** Integrator subunits are overexpressed in several EAC cell lines as compared to the non-tumorigenic esophageal cell line Het1A (representative WB). **b**, **c** INTS11 knockdown results in EAC cell growth arrest in a colony formation assay (representative assay). **d** OE33 xenografts size is significantly decreased upon INTS11 knockdown compared to the control xenografts shGFP OE33. Bars represent standard deviation (N = 6). **p < 0.01 versus CTR sample

We demonstrated that INTS11 depletion resulted in a decrease of Notch target transcription in Notch-dependent EAC cell lines OE19 and OE33 (Fig. 1c). Since Notch has a critical role in driving the neoplastic phenotype in EAC, we sought to determine if INT played a role in Notch-driven tumorigenesis of EAC. We determined that EAC cell lines OE19, OE33, FLO1, SKGT2, and EAC42 overexpressed all the INT subunits examined (INTS1, 3, 6, 9, 10, 11, 13) as compared to the non-tumorigenic esophageal cell line Het1A, indicating the importance of INT in these cancer cells (Fig. 3a). Furthermore, when we depleted INTS11 using a doxycycline-inducible shRNA vector in OE33, cells failed to form colonies in the induced state. Colonies were readily formed when the shRNA was not induced, nor was colony formation affected with an irrelevant control vector either induced or not (Fig. 3b). Similarly, when INTS11 was knocked down by siRNA in two other EAC cell lines OE19 and FLO1, the cells failed to form colonies compared to the cells transfected with control siRNA. Moreover, the non-tumorigenic Het1A cells efficiently formed colonies when transfected with either siRNA to INTS11 or siControl (Fig. 3c).

To evaluate whether INT is required for Notch-driven tumor growth in vivo, we inoculated nude mice with OE33 cells harboring a doxycycline-inducible shRNA against INTS11 and allowed tumor formation. When tumors reached > 200mm^3^, shRNA was induced by a doxycycline treatment. Over the course of the treatment, we observed that shGFP OE33 tumors (control) continued to grow and reached an average size of > 550 mm^3^ while shINTS11 OE33 tumors (2 independently derived clones) failed to grow and significantly decreased in size. By the end of the treatment, almost all mice in shINTS11 groups had no measurable tumors (Fig. 3d). Similarly, the knockdown of Notch or NACK in EAC causes attenuation of Notch signaling and cell growth arrest both in vitro and in vivo, as we have reported previously [4, 7, 18]. Together these findings indicate that the NACK-INT mode is critical for maintaining active Notch-mediated transcription to drive cell proliferation and tumorigenesis in Notch-dependent EAC.

### INTS11 depletion results in apoptosis via G2/M cell cycle arrest

**Fig. 4:**
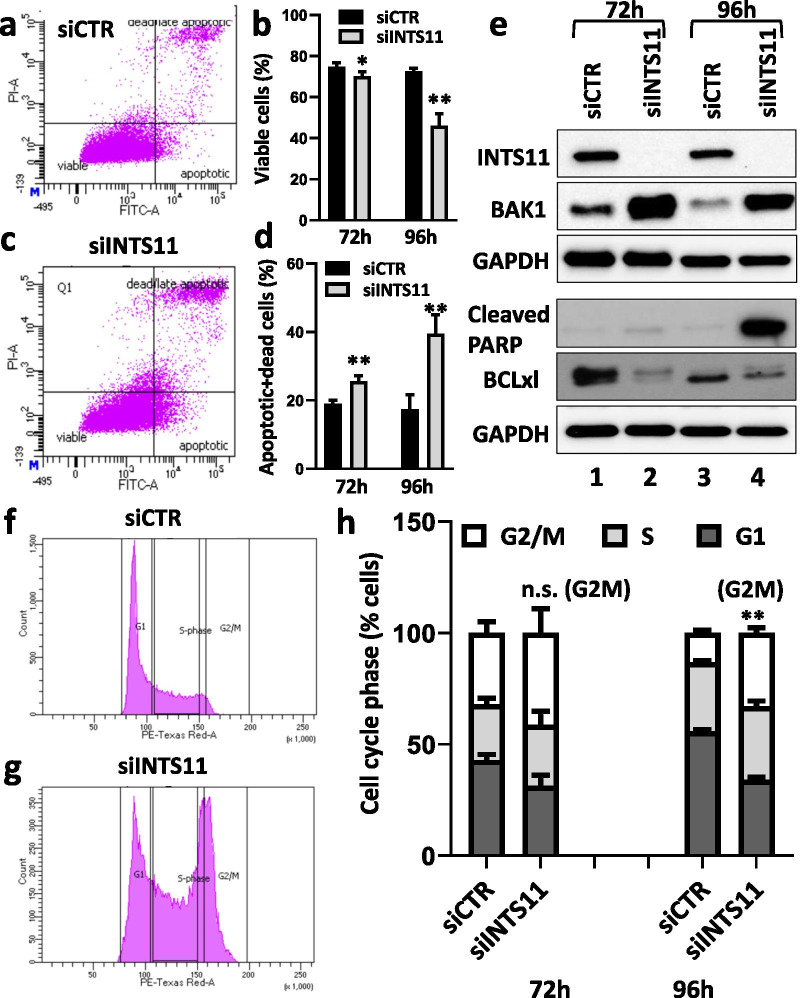
INTS11 depletion in EAC OE19 causes apoptosis. **a**–**d** INTS11 knockdown results in decreased tumor cell viability and increased apoptosis as determined by flow cytometry using PI and AnnexinV. Bars represent standard deviation (the mean from 4 biological replicates). *p < 0.05; **p < 0.01 versus CTR sample. **e** Representative WB shows an overexpression of pro-apoptotic markers BAK1 and cleaved PARP and a decreased expression of an anti-apoptotic BCL_XL_. **f**–**h** INTS11 knockdown results in G2/M cell cycle arrest as evident from the flow cytometry analysis. Bars represent standard deviation (the mean from 4 biological replicates). **p < 0.01 versus CTR sample

We demonstrated that depletion of INTS11 decreased the transcription of Notch target genes in EAC leading to tumor growth failure. As the downregulation of Notch has been associated with cell growth inhibition and stimulation of apoptosis in various types of cancer [27–29], we sought to further determine whether the INTS11 depletion results in apoptosis in EAC via flow cytometry. Under conditions of INTS11 KD in OE19 cells, there was a significant time-dependent decrease in a cell viability (Fig. 4a, b) with a concomitant increase in a number of apoptotic cells compared to the control (Fig. 4c, d). Furthermore, we observed increased levels of the proapoptotic proteins BAK1 and cleaved PARP and a decrease in the anti-apoptotic protein BCL_XL_ by western blot analysis (Fig. 4e, lanes 2 and 4). The changes in protein levels of BAK1 and BCL_XL_ (proteins of early apoptosis) began to emerge at 72 h following INTS11 KD, whereas, an increase in cleaved PARP (late apoptosis) was observed only after 96 h, indicating the proper temporal relationship between early and late apoptosis in these cells. Furthermore, we observed a block in cell cycle progression at G2/M at 96 h, with a concomitant decrease in cells in G1, indicating that cells were failing to progress through the cell cycle and arresting at G2/M resulting in apoptosis (Fig. 4f–h). When we evaluated the levels of BAK1 and BCL_XL_ as well as the cell cycle upon knockdown of Notch1, we obtained results similar to those with INTS11 knockdown. We observed an increased expression of BAK1, decreased expression of BCL_XL_, and G2/M cell cycle arrest (Additional file 5: Fig. S4). Together, these data indicate that the inhibition of Notch-dependent transcriptional regulation mediated by INTS11 KD directly contributes to the cell cycle arrest and activation of apoptosis in EAC.

## Discussion

Notch-mediated transcription is aberrantly activated in many cancers. Herein, we show that INT is a component of the Notch activation supercomplex and together with NACK is important for Notch signaling activation and tumorigenesis in esophageal adenocarcinoma. While NACK is an atypical kinase implicated in some human cancers, the Integrator complex carries out multiple biological functions including transcriptional regulation of protein-coding genes by associating with the C-terminus of RNAPII and regulating its activity [10–14, 30]. A genomic study by Beckedorff et al. described two mechanistically different roles of Integrator in the facilitation of RNAPII pause-release allowing transcriptional elongation and in the recruitment of RNAPII to the promoters depending on a type of protein-coding genes [31]. In this study, the ChIP analysis revealed that the knockdown of either NACK or INTS11 (a catalytic subunit of the INT complex) causes a decreased RNAPII-S5P ChIP signal on the Notch target promoters in EAC (Fig. 2a, e, f). However, in contrast to NACK depletion, INTS11 KD does not affect the occupancy of unmodified RNAPII indicating that INTS11 is required specifically for the activation of RNAPII while NACK is important for its recruitment to Notch target genes (Fig. 2e, g) [7].

We also discovered that NACK is required for the presence of Notch1 and INT on the Notch-dependent promoters (Fig. 2a, c). This result indicates two possibilities for the role of NACK on the Notch target promoter. The first possibility is that NACK is required for the stabilization of the NTC and RNAPII recruitment. The NTC-RNAPII complex then guides INT to the promoter and subsequently other co-factors (SPT5, CCNT1) are recruited for the transcriptional initiation. The second possibility is that NACK, in addition to stabilizing the NTC, is also required specifically for the recruitment of INT either directly or via interactions with other co-factors. Thus, further investigations are needed to determine the mechanistic details of INTS11 recruitment by NACK. In turn, INTS11 is required for the recruitment of other co-factors (SPT5, CCNT1) and activation of RNAPII that are important for transcriptional initiation.

Human homolog of SPT5 is a subunit of DSIF that can have either an inhibiting or activating role in transcription depending on its phosphorylation status. Unphosphorylated form of DSIF acts as a negative elongation factor and is involved in RNAPII pausing. Phosphorylation of DSIF by P-TEFb, whose subunits include CCNT1 and CDK9, converts the former to the positive elongation factor [23–26]. In addition, another co-factor, TFIIH-associated CDK7, phosphorylates RNAPII CTD at Ser5 [32, 33]. These key phosphorylation events promote the release of the paused RNAPII resulting in transcriptional elongation. An unexpected result was the loss of SPT5 from the promoter when INTS11 is knocked down. In previous studies it was shown that INT recruits P-TEFb to phosphorylate both DSIF and RNAPII on Ser 2 for elongation. If INT recruits P-TEFb for the RNAPII pause-release that requires phosphorylation of SPT5, then its depletion should result in the decreased CCNT1 occupancy and unchanged/increased signals for total SPT5 and RNAPII-S5P for stalled RNAPII complex. However, in our ChIP analysis, we observed a decrease of total SPT5, RNAPII-S5P, and CCNT1 upon INTS11 depletion. This result indicates that INT complex may be required not only for the recruitment of P-TEFb to phosphorylate SPT5 and RNAPII, but also for the recruitment of SPT5 itself. The result showing RNAPII-S5P decrease on the Notch target promoter when INT is depleted is in agreement with previously described role of Integrator in the facilitation of transcriptional initiation on certain types of coding genes [31]. However, since other types of genes require INT for the recruitment of RNAPII, more studies are needed to elucidate mechanistic differences allowing INT to switch between these two roles. Together, these results indicate that both NACK and INT are co-activators of Notch-mediated transcription in EAC and are required for the formation of the active Notch transcriptional supercomplex.

In this study, we also show an important role for INT in cancer as a major co-factor of Notch-mediated transcriptional regulation. Therefore, not only do we provide further mechanistic detail, but also provide a novel target for therapeutic intervention in Notch-dependent cancers. Specifically, we observed that in different Notch-dependent EAC tumor cells many of the components of INT are overexpressed as compared to the non-tumorigenic esophageal cell line Het1A. Moreover, INT is required in EAC for growth and proliferation as demonstrated by in vitro and in vivo experiments. Similar inhibitory effects in EAC were reported in our previous work upon knockdown of NACK suggesting that both INT and NACK are important for cancer cell growth in Notch-dependent EAC [4]. Furthermore, the knockdown of INT results in G2/M arrest and apoptosis, thus inhibiting tumor cell growth and proliferation. These apoptotic effects of INTS11 KD are similar to the effects observed upon the knockdown of Notch1 and consistent with previous studies with ablation of the Notch function (Fig. 4 and Additional file 5: Fig. S4) [34–36].

## Conclusions

In summary, this study identifies a novel transcriptional co-factor, the Integrator complex, that together with NACK is critical for activation of Notch-mediated transcription in esophageal adenocarcinoma. We demonstrate that NACK is important for the stabilization of the NTC on the promoter and INT recruitment, which in turn is required for the activation of RNAPII and recruitment of other transcriptional co-factors leading to gene activation. Furthermore, knockdown of INT in EAC causes G2/M cell cycle arrest and apoptosis leading to cell growth arrest both in vitro and in vivo. These findings establish the importance of NACK and the INT complex in Notch signaling activation in cancer and provide a potential novel therapeutic target.

## Supplementary Information

**Additional file 1: Table S1.** Antibodies used in ChIP and western blot experiments.

**Additional file 2: Fig. S1**. Integrator knockdown in shINTS11 OE33 clones 1-4, polyclonal population (Poly#1), and shGFP control clone after doxycycline treatment as determined by RT-qPCR. Bars represent standard deviation (N = 3).

**Additional file 3: Fig. S2**. Integrator and NACK co-localize with the NTC on Notch target genes in OE19 EAC. Representative ChIP on the *HES1*, *HES4*, and *CCND1* promoters in OE19 EAC. Bars represent standard deviation (N = 3).

**Additional file 4: Fig. S3**. INT is required for SPT5 and CCNT1 transcriptional co-factors. Representative ChIP shows that the knockdown of INTS11 results in a decrease of SPT5 and CCNT1 on the *HES1* promoter. Bars represent standard deviation (N = 3).

**Additional file 5: Fig. S4**. Notch1 depletion in EAC OE19 causes apoptosis. a. Representative WB shows an overexpression of a pro-apoptotic marker BAK1 and a decreased expression of an anti-apoptotic BCL_XL_. b. Notch1 knockdown results in G2/M cell cycle arrest as evident from the flow cytometry analysis. Bars represent standard deviation (the mean from 4 biological replicates). **p < 0.01 versus CTR sample.

## Data Availability

The LC-MS/MS datasets generated and/or analyzed during the current study are not publicly available due to the intellectual property concerns since the data were not generated with the NIH funding, but are available from the corresponding author on reasonable request. All other data generated or analyzed during this study are included in this published article.

## Abbreviations

INT: Integrator complex
NACK: Notch Activation Complex Kinase
RNAPII: RNA polymerase II
FPLC: Fast protein liquid chromatography
LC-MS/MS: Liquid chromatography-tandem mass spectrometry
ChIP: Chromatin immunoprecipitation
qPCR: Quantitative polymerase chain reaction
EAC: Esophageal adenocarcinoma
INTS11: Integrator subunit 11
KD: Knockdown
Maml: Mastermind-like
NTC: Notch Ternary Complex
ATCC: American Type Culture Collection
DMEM: Dulbecco's Modified Eagle Medium
CST: Cell Signaling Technology
CSL: CBF-1/RBPJ/Suppressor of Hairless/Lag-1
siRNA: Small interfering RNA
shRNA: Short hairpin RNA
GFP: Green fluorescent protein
HPRT1: Hypoxanthine phosphoribosyltransferase 1
RT-qPCR: Reverse transcriptase quantitative polymerase chain reaction
RIPA: Radioimmunoprecipitation assay
CCNT1: Cyclin T1
P-TEFb: Positive transcription elongation factor b
DSIF: DRB sensitivity-inducing factor
WB: Western blot
