# Supplementary figures and images for: NACK and INTEGRATOR act coordinately to activate Notch-mediated transcription in tumorigenesis
Source: Cell Commun Signal. 2021 Sep 22;19:96. doi: 10.1186/s12964-021-00776-1 (PMC8456597; doi:10.1186/s12964-021-00776-1)

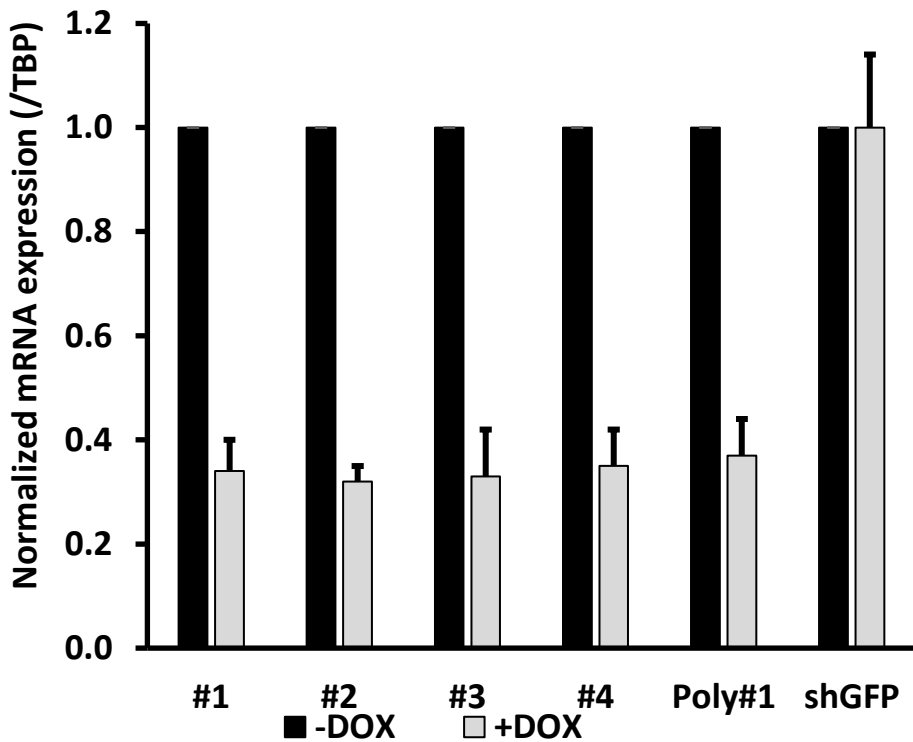

Supplement: Supplementary file 3 — Additional file 2: Fig. S1. Integrator knockdown in shINTS11 OE33 clones 1-4, polyclonal population (Poly#1), and shGFP control clone after doxycycline treatment as determined by RT-qPCR. Bars represent standard deviation (N = 3). [file 12964_2021_776_MOESM3_ESM.pdf]

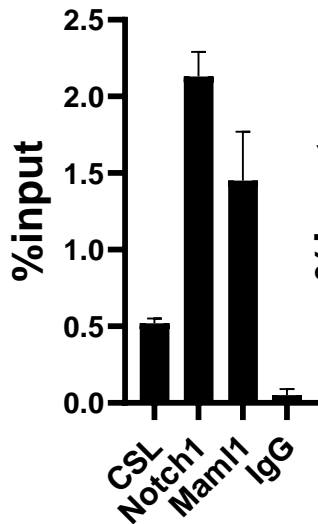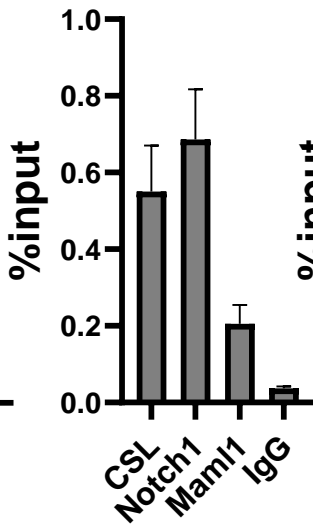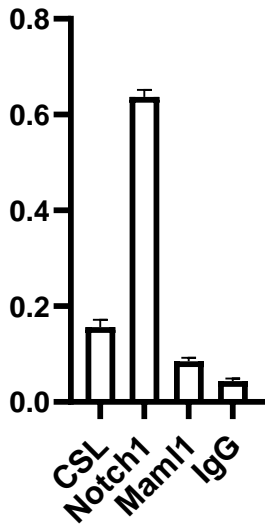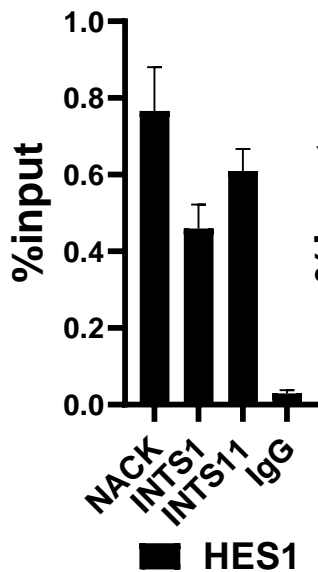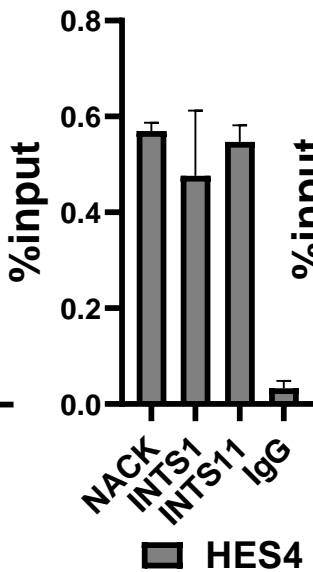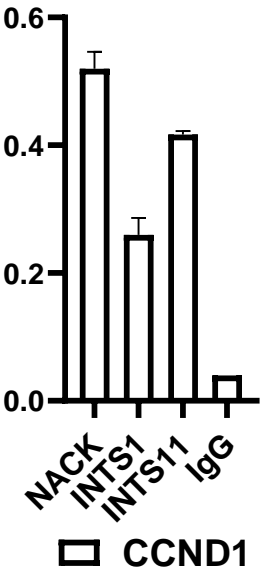

Supplement: Supplementary file 4 — Additional file 3: Fig. S2. Integrator and NACK co-localize with the NTC on Notch target genes in OE19 EAC. Representative ChIP on the HES1, HES4, and CCND1 promoters in OE19 EAC. Bars represent standard deviation (N = 3). [file 12964_2021_776_MOESM4_ESM.pdf]

**%input**

2  
1.5  
1  
0.5  
0

■ **CTR**  
■ **-INTS11**

**SPT5**      **CCNT1**      **IgG**

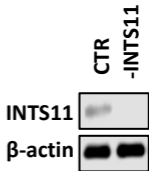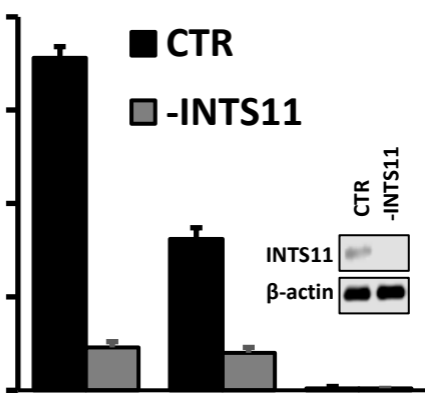

Supplement: Supplementary file 5 — Additional file 4: Fig. S3. INT is required for SPT5 and CCNT1 transcriptional co-factors. Representative ChIP shows that the knockdown of INTS11 results in a decrease of SPT5 and CCNT1 on the HES1 promoter. Bars represent standard deviation (N = 3). [file 12964_2021_776_MOESM5_ESM.pdf]

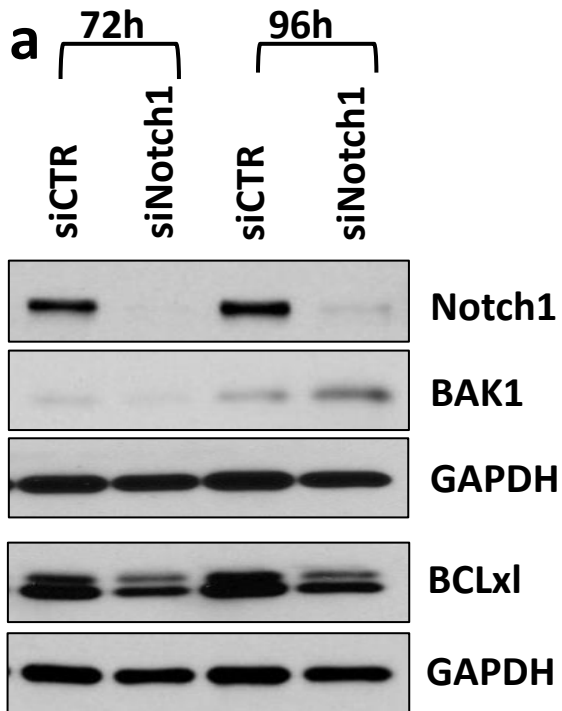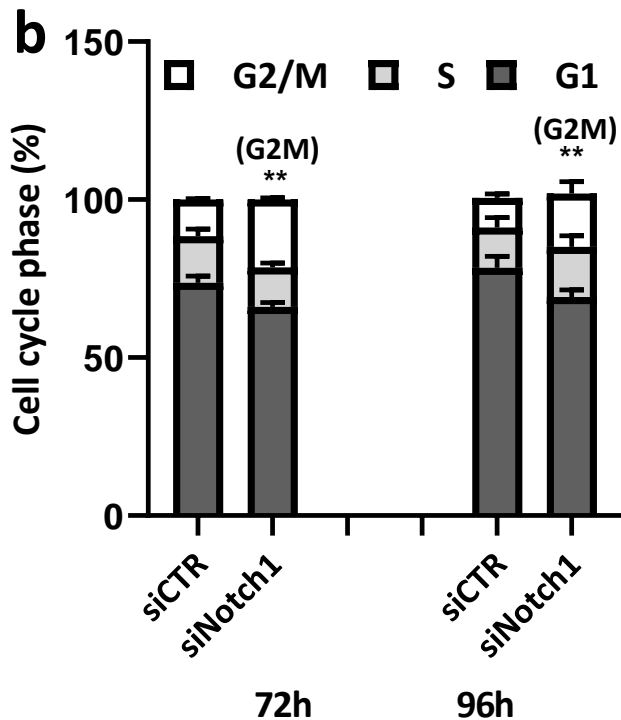

Supplement: Supplementary file 6 — Additional file 5: Fig. S4. Notch1 depletion in EAC OE19 causes apoptosis. a. Representative WB shows an overexpression of a pro-apoptotic marker BAK1 and a decreased expression of an anti-apoptotic BCLXL. b. Notch1 knockdown results in G2/M cell cycle arrest as evident from the flow cytometry analysis. Bars represent standard deviation (the mean from 4 biological replicates). **p < 0.01 versus CTR sample. [file 12964_2021_776_MOESM6_ESM.pdf]
